# Cell-based medicinal products and the development of GMP-compliant processes and manufacturing

**DOI:** 10.1186/1753-6561-5-S8-O3

**Published:** 2011-11-22

**Authors:** Luca Romagnoli, Ilaria Giuntini, Marta Galgano, Chiara Crosta, Luigi Cavenaghi, Maria Luisa Nolli

**Affiliations:** 1 Areta International s.r.l., Gerenzano (VA), 21040, Italy

## 

When performing the GMP process development and scale up of cellular therapies, a critical review of the manufacturing process and all the materials and reagents involved in the production steps is the mandatory starting point to avoid potential issues related to the quality and safety of the product. The choice of the raw materials, plastics and all the additional equipment that comes into direct contact with the product must be performed always keeping in mind that cells, as drug products, cannot be terminally sterilized. The quality of the materials and reagents utilized is therefore directly related to the quality and degree of purity of the final product. Information about the available certification must be gathered for every component and, for critical materials, audits must be performed to the manufacturing sites to qualify the supplier.

The protocols used for the cell expansion and processing (if necessary) must be designed trying to reduce at a minimum the dependence on growth factors and medium supplements. Each additional component that is added to the culture medium must be justified and its absence from the final product must be verified with validated methods. Residues that are not removed during the production steps must be accurately measured and limits must be set after performing a risk evaluation analysis, to ensure that these process-related impurities have no adverse effects on the patients.

Supplements such as Fetal Bovine Serum (FBS) are permitted for the manufacturing of cellular therapies [1], as long as the serum is sourced from a TSE-free area. Anyway, the use of a medium containing FBS should be limited to the cases for which a valid alternative could not be found. However, continuous research and development is strongly advised in order to keep up to date with the latest advances in the field of medium formulations, in order to be ready to switch to an animal-free medium as soon as it is feasible. The reduction of growth factors and supplements is also effective in controlling the manufacturing costs of a cell therapy product. An evaluation of the economical aspects and market sustainability should be performed at an early stage to increase the chances for an industrial development of the cellular product.

The manipulation steps performed during the manufacturing stage should be kept to a minimum, in order to reduce human intervention and decrease the risk of contamination. Media fill simulations must be performed in purposely stressed conditions to ensure that the process and the facility are able to support the production of a sterile product [2].

When manufacturing patient-specific therapies, extensive efforts should be directed towards the reduction in the variability of the starting material, that is usually a tissue sampled from the patient during hospitalization. Working with well-defined starting material allows for the set-up of a more robust process with comparable characteristics between batches dedicated to different patients. The specifications of the final product for parameters such as cell number, purity and potency must be wide enough to tolerate the normal biological variability of living organisms, but sufficiently narrowed down to generate comparable batches of drug. This uniformity is mandatory for the set up of clinical trials aiming at gathering a reliable analysis of the safety, tolerability and efficacy data obtained from treated patients, in order to speed up the clinical development of innovative medicinal products such as cellular therapies.
